# Spinach Methanolic Extract Attenuates the Retinal Degeneration in Diabetic Rats

**DOI:** 10.3390/antiox10050717

**Published:** 2021-05-03

**Authors:** Rocio Bautista-Pérez, Agustina Cano-Martínez, Elisa Gutiérrez-Velázquez, Martín Martínez-Rosas, Rosa M. Pérez-Gutiérrez, Francisco Jiménez-Gómez, Javier Flores-Estrada

**Affiliations:** 1 Departamento de Biología Molecular, Instituto Nacional de Cardiología “Ignacio Chávez”, Juan Badiano 1, Sección 16, Tlalpan, Ciudad de México 14080, Mexico; maria.bautista@cardiologia.org.mx; 2 Departamento de Fisiología, Instituto Nacional de Cardiología “Ignacio Chávez”, Juan Badiano 1, Sección 16, Tlalpan, Ciudad de México 14080, Mexico; agustina.cano@cardiologia.org.mx (A.C.-M.); martin.martinez@cardiologia.org.mx (M.M.-R.); 3 Laboratorio de Investigación de Productos Naturales, Escuela de Ingeniería Química e Industrias Extractivas, Instituto Politécnico Nacional, Unidad Profesional Adolfo López Mateos, Av. Instituto Politécnico Nacional S/N, Ciudad de México 07708, Mexico; elisagv57@hotmail.com (E.G.-V.); rmperezg@ipn.mx (R.M.P.-G.); 4 División de Investigación, Hospital Juárez de México, Av. Instituto Politécnico Nacional 5160, Magdalena de las Salinas, Gustavo A. Madero, Ciudad de México 07760, Mexico; microcirugiafco@hotmail.com

**Keywords:** spinach, diabetic retinopathy, advanced glycation end products, oxidative stress, inflammation, RAGE, carboxymethyl L-Lysine

## Abstract

It has been suggested that spinach methanolic extract (SME) inhibits the formation of advanced glycation end products (AGEs), which are increased during diabetes progression, so it is important to know if SME has beneficial effects in the diabetic retina. In this study, in vitro assays showed that SME inhibits glycation, carbonyl groups formation, and reduced-thiol groups depletion in bovine serum albumin incubated either reducing sugars or methylglyoxal. The SME effect in retinas of streptozotocin-induced diabetic rats (STZ) was also studied (*n* = 10) in the normoglycemic group, STZ, STZ rats treated with SME, and STZ rats treated with aminoguanidine (anti-AGEs reference group) during 12 weeks. The retina was sectioned and immunostained for Nε-carboxymethyl lysine (CML), receptor RAGE, NADPH-Nox4, inducible nitric oxide synthase (iNOS), 3-nitrotyrosine (NT), nuclear NF-κB, vascular endothelial growth factor (VEGF), glial fibrillary acidic protein (GFAP), S100B protein, and TUNEL assay. Lipid peroxidation was determined in the whole retina by malondialdehyde (MDA) levels. The results showed that in the diabetic retina, SME reduced the CML-RAGE co-localization, oxidative stress (NOX4, iNOS, NT, MDA), inflammation (NF-κB, VEGF, S100B, GFAP), and apoptosis (*p* < 0.05). Therefore, SME could attenuate the retinal degeneration by inhibition of CML–RAGE interaction.

## 1. Introduction

Plant-based diets have been associated with a lower risk of developing chronic diseases such as obesity, type 2 diabetes, and coronary heart disease. Spinach (*Spinacia oleracea* L.) is an edible leafy vegetable consumed throughout the world due to providing an appreciable amount of fibers, vitamins, and minerals [1,2]. Several of its phytochemicals have been identified, including chlorophylls, carotenoids (lutein and zeaxanthin) [3], and phenolic compounds (flavones, flavonoids, coumarins) [4,5,6,7,8] (see supplement 1).

It has been reported that spinach, when ingested as a food, water-soluble extract, or in a freeze-dried form, and its thylakoids have anti-cancer, anti-obesity, and hypoglycemic properties [9]. The anti-inflammatory effect of spinach has been demonstrated in animal endotoxemia models, obese animals, and healthy humans [9]. Its antioxidant effect was studied in human prostate carcinoma cells, animal models with obesity, UV-irradiated mice, and adenocarcinoma of the transgenic mouse prostate [9,10]. The anti-glycation and anti-inflammatory effects of spinach methanolic extract (SME) have been studied in the model of glucose-induced diabetes in zebrafish and isoproterenol-induced myocardial necrosis in rats, respectively [11,12]. SME diminished the glycosylated hemoglobin and fructosamine levels, including the glycated protein levels, by reducing aldose reductase activity in lens ocular [11]. The above findings suggest that SME might have a preventive effect in the progression of diabetes mellitus complications as diabetic retinopathy (DR).

DR progressively leads to loss of visual acuity or blindness that has been related to inflammation, oxidative stress, and accumulation of advanced glycation end products (AGEs) [13,14]. The AGEs induce protein crosslinking, altering structure/function and its turnover/clearance. AGEs can be produced by a non-enzyme reaction between the glucose with a free amino group of proteins, forming reversible intermediates as Schiff’s bases and Amadori products (fructosamine) [15]. Other mechanisms for AGEs formation include the “carbonyl stress” pathway, where oxidation of sugars and lipids create dicarbonyl intermediate compounds as glyoxal and methylglyoxal (MGO), which lead to AGEs formation as the Nε-carboxymethyl lysine (CML) [16]. Increased CML levels in serum and vitreous can be a biochemical marker for both the appearance and progression of DR [17,18]. In the retina, the activation of receptor RAGE by AGEs (AGEs-RAGE) induces the overexpression of glial fibrillary acidic protein (GFAP) and pro-inflammatory factors, regulated by the nuclear factor kappa-light-chain-enhancer of activated B cells (NF-κB) [19]. NF-κB positively regulates RAGE expression by acting as a positive feedback mechanism [20]. On the other hand, high concentrations of S100B, a calcium-binding protein, may also activate NF-κB, inducing neuroinflammation and glial cell activation [21]. The overexpression of S100B in astrocytes provokes neurotoxic effects manifested by retinal astrogliosis [22].

Strategies have been experimentally developed to prevent retinal damage. Some phytochemicals of spinach isolated from other plants have been associated with inhibition of AGEs and oxidative stress [8,23,24,25,26]. Lutein, astaxanthin, and kaempferol have protected human-derived retinal pigment epithelial cells (ARPE-19) from damage through their anti-AGEs, antioxidant, and anti-apoptosis properties [26,27,28]. These results suggest that phytochemicals of spinach have a relevant role in the prevention of retinal degeneration like DR. Another strategy has been using aminoguanidine (AG) as a potent inhibitor of glycation and iNOS activity, attenuating the CML accumulation and formation of reactive di-carbonylic precursors in rats [29]. However, in a multicentric and randomized clinical trial with 690 diabetic patients, the beneficial effect of AG was not established clearly [30].

The changes in retinal degeneration under hyperglycemic conditions have been scarcely studied. Therefore, it is interesting to know if SME protects retinal layers from damage related to its anti-AGEs, antioxidant, and anti-inflammatory properties in the retina of streptozotocin-induced diabetic rat.

## 2. Materials and Methods

### 2.1. Preparation of the Methanolic Extract of Spinach (SME)

The fresh leaves of spinach (*S. oleracea* L.) were harvested in the autumn–winter season in an agricultural field of the Puebla Province, México, and identified by a botanist in the herbarium of The National Polytechnic Institute (IPN, México City, Mexico). Voucher specimen number 4532 was deposited in the herbarium of the National School of Biological Sciences of IPN.

The leaves were dehydrated and finely ground. The dried leaves were macerated with methanol at room temperature, filtered through cellulose filters (Whatman^®^, Maidstone, UK), and dried using a rotatory vacuum evaporator (BUCHI Rotavapor R-200, Switzerland) at 45 °C to yield a green gum (95.0 g/kg dry leaves). The SME was stored in the dark at 4 °C until use. For all assays, the extract was reconstituted in distilled water [11].

### 2.2. In Vitro Assays of SME Anti-Glycation Activity

#### 2.2.1. Formation of Advanced Glycation End Products Derived from Bovine Serum Albumin (BSA-AGEs)

The BSA-AGEs assay was carried out, as described previously [31]. Briefly, 10 mg/mL BSA (fraction V; Sigma-Aldrich, St. Louis, MO, USA) was added either in D-glucose 0.5 M, D-fructose 0.5 M, or methylglyoxal 2.5 mM, and made in a solution 0.1 M PBS (pH 7.4) and 0.02% sodium azide. SME concentrations (5, 10, 25, 50, 100, and 200 mg/mL) were added to each mixture and then incubated at 37 °C for 4 weeks. Afterward, the unreacted sugars were removed by dialysis against distilled water for two days at 4 °C. BSA-AGEs levels were determined by fluorescence spectrophotometry (Ex 370/Em 440 nm; model LS 30, PerkinElmer LAS Ltd., Buckinghamshire, UK) [32]. The glycation of BSA protein by reducing sugars and MGO were the positive controls (BSA glycated), while the incubation of BSA glycated with aminoguanidine (1 mg/mL) was used as the negative control (BSA glycated-AG). The assays were performed in triplicate.

#### 2.2.2. Fructosamine Assay

The fructosamine level was determined by the nitro blue tetrazolium (NBT) assay [33], which is based on the ability of fructosamine to reduce NBT, forming formazan, a colored end-product under alkaline conditions. Ten microliters either of the controls or glycated BSA-incubated SME concentrations (BSA-SME) were added in 90 µL of NBT at 2.5 mM, prepared in a carbonate buffer (0.1 M; pH 10.3); all were incubated at 37 °C for 30 min. The absorbance at 530 nm was measured. Fructosamine concentrations (nmol/mg) were calculated according to a standard curve of formazan.

#### 2.2.3. Determination of Carbonyl Groups Formation and Thiol Groups Depletion in BSA-AGEs

Carbonyl group concentration was measured in BSA-SME and controls samples, according to Levine et al. [34]. A solution of 2,4-dinitrophenylhydrazine (DNPH; 10 mM) in 400 μL of 2.5 M HCl was added to 100 μL each sample and incubated in darkness for 60 min at room temperature. Then, 500 μL of trichloroacetic acid (20% *w*/*v*) was added and kept on ice for 5 min. The samples were centrifuged at 4000 rpm for 15 min, and the protein pellet was washed three times with ethyl acetate/ethanol (1:1 *v*/*v*). Afterward, the samples were suspended in 250 μL guanidine hydrochloride to 6 M. The concentration of carbonyl groups (nmol/mg protein) was calculated by spectrophotometry at 370 nm absorbance (EON, BioTek Instruments, Inc., Winooski, VT, USA) using the absorption coefficient of DNPH (22,000 M^−1^ cm^−1^).

According to Ellman’s method, the determination of thiol group depletion in BSA-SME and control samples was performed. Briefly, 10 µL of 5,5′-Disulfanediylbis(2-nitrobenzoic acid) (DNTB; 10 mM, prepared in PBS) with 50 µL of glycated samples was incubated for 15 min at room temperature, then the absorbance at 410 nm was measured. The level of free thiol groups was calculated using a standard curve of L-cysteine (0.4–11 μM) and expressed in nmol/mg protein [34].

### 2.3. Effect of SME on the Retina of Diabetic Rats

#### 2.3.1. Diabetes Induction in the Rats

Wistar male rats weighing 250 ± 10 gr (N = 40) fasted for 8 h were used. A dose intraperitoneal of streptozotocin (60 mg/kg) in citrate buffer (10 mM, pH 4.5) (Sigma-Aldrich, Inc., St. Louis, MO, USA) was administered [35]. Three days later, the glucose level was measured (glucometer; ACCU-CHEK active; Roche Diagnostics, GmbH, Mannheim, Germany) by collecting a drop of a blood sample from the tail. Animals with glycemic levels higher than 350 mg/dL were recruited for the study. The glucose in the blood was measured weekly for 12 weeks.

#### 2.3.2. Experimental Design

The streptozotocin-induced diabetic rats (STZ) were divided randomly into the following groups (*n* = 10): STZ treated intragastrically with 2 mL of drinking water (STZ); STZ treated with SME at 400 mg/kg (STZ-SME); normoglycemic rats (NG); and STZ treated with 50 mg/kg aminoguanidine (AG; Sigma-Aldrich, Inc.) (STZ–AG). STZ–AG was used as an anti-AGE reference group. The assigned doses were administered every 24 h (9:00 a.m.) for 12 weeks. The glycemic level in all groups was monitored weekly.

The SME dosage regimen at 400 mg/kg was based on the following: 7 g of SME is obtained from 100 g of fresh spinach (data not shown). This amount is equivalent to the daily consumption of an average person of 70 kg in the American diet [36], which corresponds to 100 mg of extract/kg of body weight. On the other hand, due to the difference in the accelerated metabolism of rats, it is recommended to increase the consumption of any natural extract up to 6.4 times for comparison studies with humans [37]. The dose of 400 mg/kg that we used was based mainly on the results obtained in a model of myocardial necrosis induced in Wistar rats, which showed that the anti-inflammatory effect of SME is more significant at 300 mg/kg [12].

#### 2.3.3. Histological and Immunohistochemistry Evaluations

Enucleated eyes were fixed in neutral formalin and then were dehydrated in graded alcohols and embedded in paraffin. Histological sections of 2 μm were mounted on electrocharged slides, dewaxed, and rehydrated up to antigen recovery solution (K035; 10× citrate Buffer, pH6; Diagnostic BioSystems, Pleasanton, CA, USA). A polymer-based immunodetection system (PolyVue^®^ mouse/rabbit DAB detection system, Diagnostic BioSystems) was used. The following primary antibodies were used: glial fibrillary acidic protein (anti-GFAP; MAB360; Chemicon International, Inc., Temecula, CA, USA); vascular endothelial grown factor (anti-VEGF; sc-7269; Santa Cruz Biotechnology, Inc., Dallas, TX, USA), S100 calcium-binding protein B (anti-S100B; ab52642; Abcam PLC, Cambridge, UK), and nuclear factor kappa-light-chain-enhancer of activated B cells (anti-NF-κB p65; sc-8008). All dilutions were at 1:200 and incubated overnight at 4 °C. A secondary antibody (Mouse/Rabbit PolyVue™) was added according to the supplier’s instructions. Sections were stained with DAB plus/chromogen substrate and hematoxylin. Histological observations and the capture of images were carried out in a Carl Zeiss microscope (Carl Zeiss Microscopy GmbH, Jena, Germany).

For immunofluorescence staining, the slides were incubated overnight at 4 °C with following primary antibodies: carboxymethyl Lysine (anti-CML; ab124145; Abcam PLC, Cambridge, UK), AGE receptor (anti-RAGE; sc-365154), NADPH oxidase 4 (anti-Nox4; ab133303), 3-nitrotyrosine (anti-NT; ab110282), and nitric oxide synthase (inducible) (anti-iNOS; A00368-1; Boster Bio, Pleasanton, CA, USA). Then, the following secondary antibodies were used: for anti-RAGE, goat anti-mouse IgG (FITC)-conjugated (1:200; Santa Cruz, Biotechnology, Inc.); for anti-CML and anti-NT, mouse anti-rabbit IgG–PE (SC-3753); and for anti-iNOS and anti-Nox4, m-IgGkBP-PE (Sc-516141). All were incubated at room temperature for 60 min. Afterward, sections were mounted in a medium containing 4′,6-Diamidine-2′-phenylindole dihydrochloride (DAPI). Anti-CML and anti-RAGE antibodies were co-hybridized in the same slide. FLoid™ Cell Imaging Station (Life technologies Carlsbad, San Diego, CA, USA) was used for fluorescence images.

#### 2.3.4. Apoptosis Evaluation

The apoptosis of retinal cells was detected according to the manual described by terminal deoxynucleotidyl transferase (TdT)-mediated deoxyuridine triphosphate (dUTP) nick-end labeling (TUNEL) assay using the In Situ Cell Death Detection Kit, TMR (tetramethyl-rhodamine-5-dUTP) red, version 12 (12156792910; Roche Diagnostics). Briefly, the dewaxed slides were rehydrated and rinsed twice with PBS. Then, they were incubated with the TUNEL reaction mixture in a humidified atmosphere for 60 min at 37 °C. Afterward, sections were mounted with DAPI and observed in fluorescence microscopy (FLoid™ Cell Imaging Station). IOD for TUNEL was calculated for retinal layers.

#### 2.3.5. Lipid Peroxidation Assay

For the evaluation of malondialdehyde (MDA) levels in the retinal tissue, approximately 3 mg fresh tissue (*n* = 3) were homogenated and processed as previously reported [38]. MDA levels were quantified following the kit supplier’s instructions (OXItek-TBARS assay kit, Enzo Life Sciences, Farmingdale, NY, USA).

### 2.4. Statistical Analysis

For statistical analysis, all retinal micrographs were captured with a magnitude of 200× at 100 µm beyond the optic nerve region. Approximately 40 images per group of animals (*n* = 7) were analyzed. Image analysis was performed with software Image-Pro Premier Version 9.0 (Media Cybernetics, Inc., Rockville, MD, USA).

We used GraphPad Prism software (La Jolla, CA, USA; version 8.0). The figures show values graphed in box-and-whisker plots (median, first-third quartile, minimum-maximum value) for the ganglion cell layer (GCL), inner nuclear layer (INL), and outer nuclear layer (ONL). The mean and standard deviation (mean ± SD) are shown in Appendix A (Table A1, Table A2 and Table A3). In all cases, a one-way ANOVA test followed by Tukey’s test was performed. *p* <0.05 was considered statistically significant.

## 3. Results

### 3.1. Methanolic Extract of Spinach (SME) Inhibits Bovine Serum Albumin (BSA) Glycation In Vitro

#### 3.1.1. Effect of SME on the Formation of Advanced Glycation End Products (AGEs) and Fructosamine

The antiglycation effect at different concentrations of SME on BSA was determined. This effect was evaluated by inhibition of formation of AGEs and fructosamine in the mixtures: BSA-reducing sugars (glucose or fructose) and BSA-methylglyoxal (BSA-MGO) after four weeks of incubation. Table 1 shows the results of AGEs formation evaluated by fluorescence. In the BSA-reducing sugars and BSA-MGO mixtures incubated with SME at 200 mg/mL (SME-200), the level of AGEs decreased compared to the positive controls (BSA-glucose, BSA-fructose, and BSA-MGO) (*p* < 0.05). Concentrations below 200 mg/mL did not show significant differences compared to positive controls. The inhibition of AGEs formation by aminoguanidine (AG) in BSA-reducing sugars and BSA-MGO was higher than SME-200 (*p* < 0.05) and, therefore, than the positive controls (*p* < 0.01). Both SME-200 and AG decreased fructosamine concentrations compared with controls (*p* < 0.05). SME-200 compared to AG in incubation with BSA-MGO further reduced the level of fructosamine (*p* < 0.05) (Table 1).

#### 3.1.2. Effect of SME on Levels of Carbonyl and Reduced-Thiol Groups

To determine if SME inhibits the BSA oxidation related to the formation of carbonyl groups and depletion of thiol groups during its glycation, in vitro assays were carried out. Table 1 shows levels of the carbonyl groups under incubation conditions using the mixtures of BSA-reducing sugars and BSA-MGO. In these mixtures, there was an inverse relation between SME concentrations and the level of carbonyl groups. In particular, SME-200 induced a significant diminishing compared to the positive controls (*p* < 0.05). As expected, AG significantly inhibited the formation of carbonyl groups in the BSA-reducing sugars and BSA-MGO (*p* < 0.01), demonstrating its inhibitory effect on protein carbonylation, as reported [39]. Between SME and AG, there was no difference in carbonylation level.

Determination of thiol group levels in BSA was performed during the glycation assay. SME concentrations showed a tendency to conserve thiol groups in all mixtures. In SME-200, a significant augment in the level of thiol groups was found in BSA-reducing sugars (*p* < 0.05) and the BSA-MGO (*p* < 0.01) compared to positive controls. AG induced the highest conservation of thiol groups in BSA-reducing sugars and BSA-MGO (*p* < 0.05). However, in the incubation of SME-200 with BSA-MGO, thiol group levels are higher compared to AG (*p* < 0.05) (Table 1).

Concentrations of 5, 10, and 25 mg/mL of SME studied in a previous assay did not show significant levels for the inhibition of AGEs, fructosamine, carbonyl group formation, and depletion of thiol groups of the BSA (data not shown).

In supplement 2, the probable carbonyl and AGEs compounds produced during incubation of BSA with the reducing sugar are shown. The molecules resulting from the transformation of the reduced-thiol group (cys34) of BSA produced by MGO or glyoxal incubation are also shown [40,41,42].

### 3.2. Studies of SME in the Retina of STZ-Induced Diabetic Rats (STZ)

#### 3.2.1. Effect of SME on Carboxymethyl-Lysine (CML) Formation and Its Interaction with the AGEs Receptor (RAGE) in STZ

**Figure 1:**
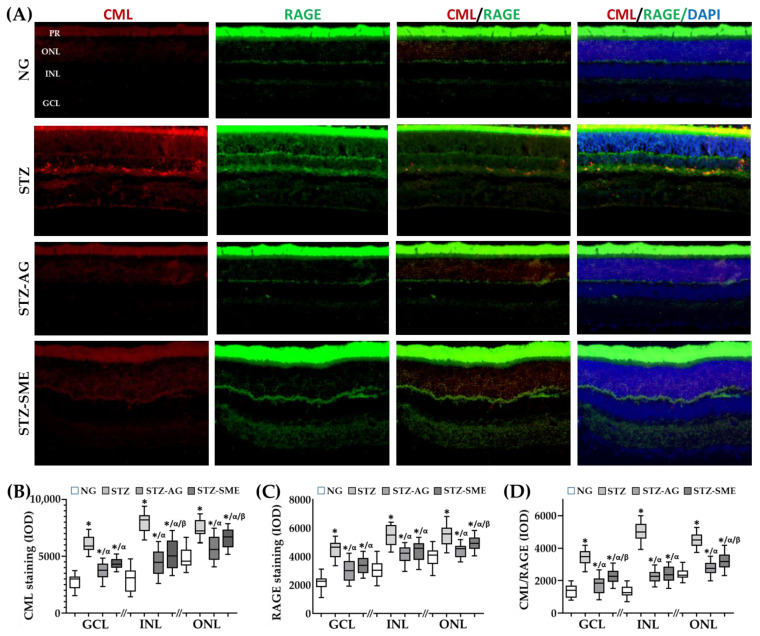
Effect of spinach methanolic extract (SME) on carboxymethyl-lysine (CML), receptor for advanced glycation end products (RAGE), and CML–RAGE interaction (CML/RAGE) in the retina of streptozotocin-induced diabetic rats (STZ). (**A**) The distribution for CML, RAGE, and CML/RAGE stainings are shown in retinas of normoglycemic rats (NG), STZ, treated with aminoguanidine (STZ-AG), and treated with SME (STZ-SME). (**B**–**D**) show the integrated optical density (IOD, lum/pixel^2^) in a box-and-whisker plot in the ganglion cell layer (GCL), inner nuclear layer (INL), and outer nuclear layer (ONL) for each immunostaining. * *p* < 0.01 compared to NG; α *p* < 0.01 compared to STZ; and β *p* < 0.01 compared to STZ-AG. Magnification 200×. *n* = PR = photoreceptors.

Figure 1 shows the effects of SME at 400 mg/kg on the CML formation, RAGE expression, and CML–RAGE interaction (CML/RAGE) on the retina of STZ. In Figure 1A, it is observed that both CML and RAGE are distributed in the ganglion cell layer (GCL), internal cell layer (INL), and outer nuclear layer (ONL), whereas CML/RAGE merged was mainly distributed in INL. Figure 1B–D shows the integrated optical density (IOD) for CML, RAGE, and CML/RAGE in the different layers of the retina (presented as a minimum to maximum box-and-whisker plot). The data (mean ± SD) are included in Appendix A (Table A1).

Figure 1B shows that SME decreased the CML immunostaining in all three retinal layers compared to STZ (*p* < 0.01). The treatment with AG (STZ-AG) presented a more significant decrease than STZ-SME in CML staining in both INL and ONL (*p* < 0.05). In GCL, there was no difference. In NG, CML staining was lower in the three retinal layers than all those treated with STZ (*p* ≤ 0.05). Figure 1C,D show that stainings for RAGE and CML/RAGE were higher in STZ than STZ-SME, STZ-AG, and NG (*p* < 0.001).

Regarding RAGE staining between STZ-SME and STZ-AG, there was no difference between GCL and INL, whereas in ONL, an increase in STZ-SME was observed (*p* < 0.01). STZ-SME decreased CML/RAGE in all three retinal layers compared to STZ (*p* < 0.01), whereas STZ-AG in GCL and ONL induced a more significant decrease than SME (*p* < 0.01). In INL, between STZ-AG and STZ-SME, there was no difference.

#### 3.2.2. Antioxidant Effect of SME in the Retina of STZ

**Figure 2:**
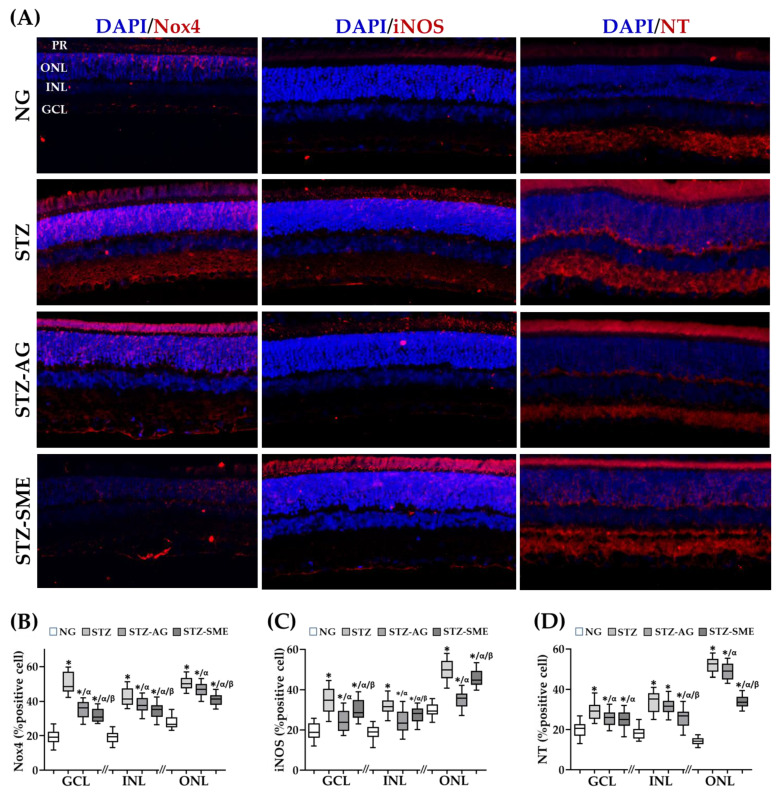
Antioxidant effect of SME in the retina of STZ. (**A**) Representative micrographs of immunofluorescence for NADPH oxidase 4 (Nox-4) (**A**), inducible nitric oxide synthase (iNOS), and nitrotyrosine (NT). (**B**–**D**) show the box-and-whisker plots of the percentage positive cells in GCL, INL, and ONL for each immunostaining in NG, STZ, STZ-AG, and STZ-SME groups. The nuclei were stained with DAPI (blue). * *p* < 0.01 compared to NG; α *p* < 0.05 compared to STZ; and β *p* < 0.01 compared to STZ-AG. Magnification 200×. In supplement 3, DAPI and each specific antibody stain are shown in a single-channel and the fused images.

The antioxidant effect of SME was evaluated by immunostaining for NADPH oxidase-4 (Nox-4), inducible nitric oxide synthase (iNOS), and nitrotyrosine (NT). Figure 2A shows the distribution of each immunostaining in the three layers of the retina (GCL, INL, and ONL). Figure 2B–D shows the percentage of the positive cells for each marker presented as a minimum to maximum box-and-whisker plot. The data (mean ± SD) are included in Appendix A (Table A2). For Nox4 staining (Figure 2B), the SME treatment (STZ-SME) reduced the percentage of cells stained in the three retinal layers compared to STZ (*p* < 0.05). Comparing STZ-SME with STZ-AG, STZ-SME showed a lower decrease for Nox4 in the three retinal layers (*p* < 0.05).

The iNOS immunostaining was also distributed in the three retina layers (Figure 2A). In Figure 2C, STZ-SME decreased the percentage of positive cells for iNOS in the three retinal layers compared to STZ (*p* < 0.01). Comparing SME effect with AG in this immunostaining, STZ-AG decreased significantly in the three retinal layers (*p* < 0.01). Figure 2D shows the percentage of the positive cells for NT in the different groups. SME decreased this value for all retinal layers compared to STZ (*p* < 0.01). When the percentage between SME and AG was compared, there was no difference in GCL, whereas in INL and ONL, STZ-SME had a lower percentage (*p* < 0.01). The NG group showed the lowest oxidative markers than STZ groups (*p* < 0.01).

**Figure 3:**
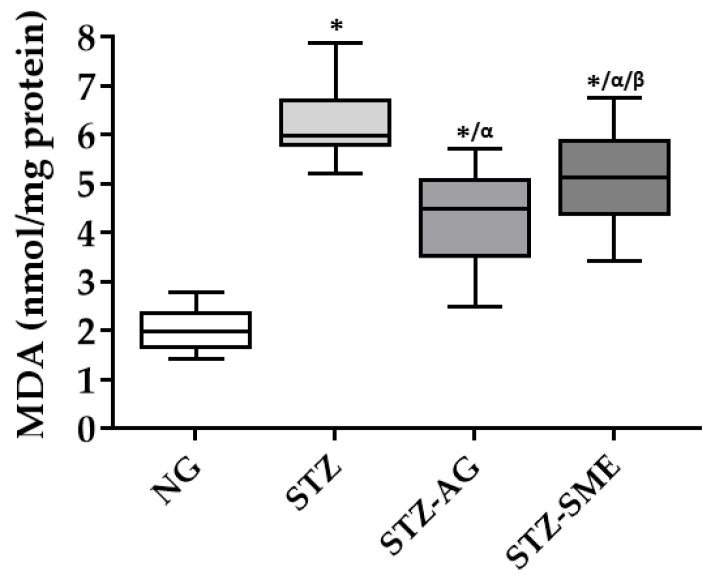
Antioxidant effect of SME in the diabetic retina determined by lipid peroxidation. Malondialdehyde (MDA) levels in the NG, STZ, STZ-AG, and STZ-SME groups were analyzed. Concentrations are shown in the box-and-whisker plot. * *p* < 0.01 compared to NG; α *p* < 0.01 compared to STZ; and β *p* < 0.05 compared to STZ-AG.

The antioxidant effect of SME in the diabetic retina by malondialdehyde (MDA) formation levels was also determined. Figure 3 shows the MDA concentrations in the groups studied. In STZ-SME and STZ-AG, MDA levels (5.1 ± 0.1 and 4.34 ± 1.0) decreased compared to STZ (6.3 ± 0.8; *p* < 0.01). Comparing STZ-SME with STZ-AG, the levels are increased in STZ-SME (*p* < 0.05). In NG (2.07 ± 0.42), the levels were lowest compared to the STZ groups (*p* < 0.01).

#### 3.2.3. Effect of SME on the Retinal Inflammation of STZ

The staining percentages of nuclear NF-κB p65, vascular endothelial growth factor (VEGF), and glial fibrillary acidic protein (GFAP) were evaluated to determine if SME treatment in STZ attenuates retinal inflammation.

**Figure 4:**
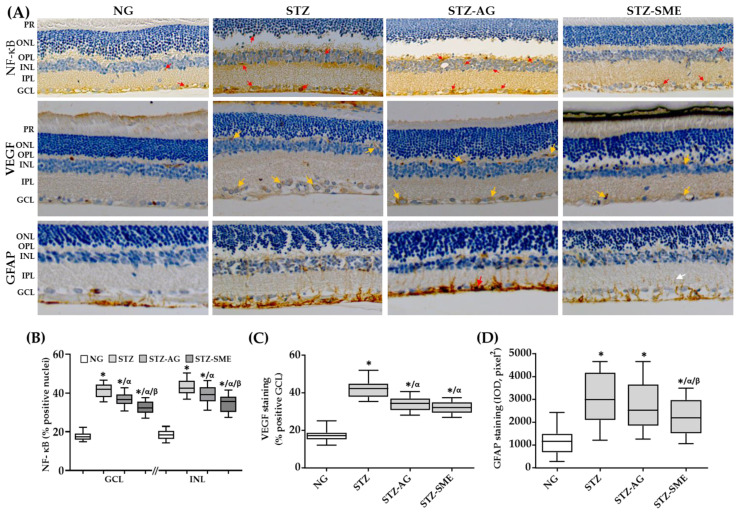
Effect of SME in the retinal inflammation of STZ. (**A**) Representative images of the immunostaining for nuclear NF-κB (represented by red arrows), vascular endothelial growth factor (VEGF; represented by the yellow arrows), and glial fibrillary acidic protein (GFAP; red and white arrows indicate maximum and minimum staining intensity, respectively) in the NG, STZ, STZ-AG, and STZ-SME groups. (**B**,**C**) Box-and-whisker plots show the percentage of positive staining for nuclear NF-κB (GCL, INL, and ONL) and VEGF (in the GCL), respectively. In (**D**), the IOD for GFAP staining along the retina is shown. * *p* < 0.01 compared to NG; α *p* < 0.01 compared to STZ; and β *p* < 0.01 compared to STZ-AG. Magnification 200×.

The nuclear staining of NF-κB was located in the three retinal layers (GCL, INL, and ONL) in all groups (Figure 4A). STZ-SME presented a lower percentage of NF-κB staining compared to STZ and STZ-AG in the three retinal layers (*p* < 0.01) (Figure 4B). Similarly, diabetic rats treated with AG (STZ-AG) showed that NF-κB staining is decreased in all three layers of the retina compared to STZ (*p* < 0.05). The NG group showed the lowest values of the percentage of NF-κB staining in the three layers of the retina (*p* < 0.01). The data (mean ± SD) are included in Appendix A (Table A3). Retinal VEGF staining (Figure 4A) and the percentage of positive cells were evaluated in the experimental groups (Figure 4C). VEGF staining was mainly localized to GCL (Figure 4A). In STZ, the percentage of VEGF positive cells is increased (42.1 ± 4.42) compared to SME (32.28 ± 3.1; *p* < 0.01), AG (34.18 ± 3.46; *p* < 0.01), and NG (17.32 ± 2.94; *p* < 0.01). However, there was no significant difference between SME and AG. The percentage of VEGF staining in the NG group was the lowest of all groups (*p* < 0.001).

Retinal inflammation was also determined by GFAP staining as a marker of astrogliosis (Figure 4A), and its distribution along the layers of the retina was quantified by IOD (Figure 4D). In the retina of STZ, the presence of GFAP was observed from GCL to INL (IOD = 3126 ± 1018), while in the NG group, GFAP was observed mainly in the nerve fiber layer (IOD = 1160 ± 561; *p* < 0.01). In STZ-SME, GFAP staining was decreased (IOD = 2192 ± 752) compared to STZ (*p* < 0.01) and compared to STZ-AG (IOD = 2796 ± 995; *p* < 0.0018). However, there were no significant differences between STZ and AG.

#### 3.2.4. SME Effect on the S100 Calcium-Binding Protein B (S100B) Levels in the Retina of STZ

**Figure 5:**
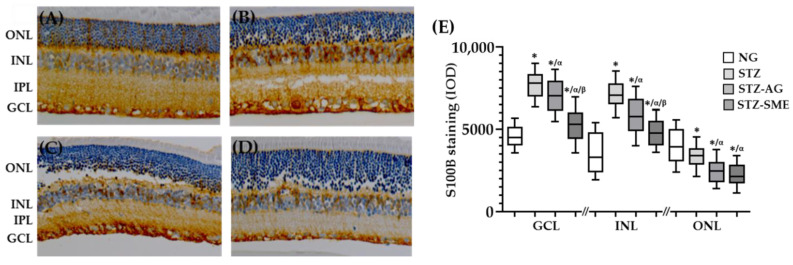
The S100 calcium-binding protein B (S100B) levels in the retina of the STZ. Retina sections were stained with anti-S100B in the NG (**A**), STZ (**B**), STZ-AG (**C**), and STZ-SME (**D**) groups. (**E**) Shows the box-and-whisker plot of the IOD for S100B protein staining in the three retinal layers for all groups. * *p* < 0.01 compared to NG; α *p* < 0.01 compared to STZ; and β *p* < 0.01 compared to STZ-AG. Magnification 200×.

The S100B protein is related to retinal inflammation regulated by the nuclear internalization of NF-κB. Hence, to investigate if SME affects S100B expression in the retina of STZ, immunostaining (IOD) for this protein was carried out. In all groups, the S100B staining was distributed from GCL to ONL (Figure 5A). Figure 5B shows that in NG, the IOD of S100B staining was the highest in ONL compared to treated groups (*p* < 0.01), but were lower in GCL and INL (*p* < 0.01). In SME, the IOD of S100B staining was decreased in the three layers compared to STZ (*p* < 0.01 for all layers). In SME, the S100B staining in GCL and INL were lower than AG (both, *p* < 0.01); however, in ONL, there was no difference. The data (mean ± SD) are included in Appendix A (Table A3).

#### 3.2.5. SME Effect on the Retinal Degeneration in STZ

The protective effect of SME in the retinal degeneration of GCL, INL, and ONL of STZ was evaluated by terminal deoxynucleotidyl transferase (TdT)-mediated deoxyuridine triphosphate (dUTP) nick-end labeling (TUNEL) assay.

**Figure 6:**
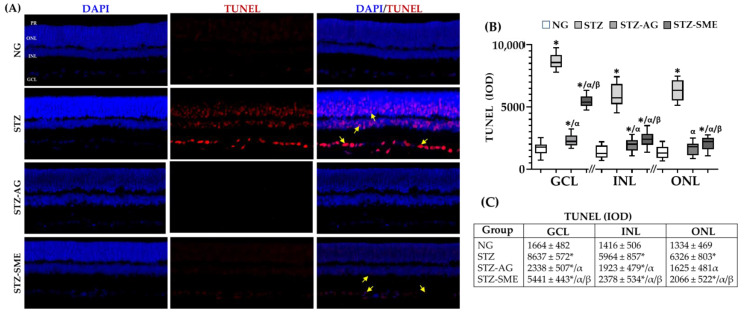
Effect of SME on the retinal degeneration of STZ. Apoptosis was determined by terminal deoxynucleotidyl transferase (TdT)-mediated deoxyuridine triphosphate (dUTP) nick-end labeling (TUNEL) assay in the NG, STZ, STZ-AG, and STZ-SME groups (**A**). Yellow arrows indicate representative cell apoptosis (**A**). The nuclei were stained with DAPI (blue). (**B**) The box-and-whisker plot of the IOD for the TUNEL staining in GCL, INL, and ONL. (**C**) Data shown in mean ± SD. * *p* < 0.01 compared to NG; α *p* < 0.01 compared to STZ; and β *p* < 0.01 compared to STZ-AG. Magnification 200×.

Figure 6A shows the representative images of the apoptosis distribution in the retinal of the groups studied (NG, STZ, STZ-AG, and STZ-SME). Figure 6B shows a box-and-whisker plot of the IOD for TUNEL staining in GCL, INL, and ONL. In STZ-SME and STZ-AG, apoptosis is significantly diminished in all layers compared to STZ (*p* < 0.01). STZ-AG had a more significant decrease in apoptosis compared to STZ-SME (*p* < 0.01). In the three retinal layers of NG, the TUNEL staining is lower than those treated with STZ (*p* < 0.01). In ONL, STZ-AG and NG did not show significant differences. The data (mean ± SD) are included in Figure 6C.

## 4. Discussion

This study reports the beneficial effects of the spinach methanolic extract (SME) in retinal layers of diabetic rats related to its anti-advanced glycation end products (AGEs), antioxidant, and anti-inflammatory properties. SME has been shown to decrease protein glycation, reducing glycosylated hemoglobin, fructosamine, and aldose reductase in zebrafish with induced diabetes, suggesting an anti-glycation activity [11]. Our in vitro experiments using bovine serum albumin (BSA) demonstrated that SME reduced the formation of AGEs, fructosamine, carbonyl groups, and the depletion of reduced thiol groups in BSA, incubated with either reducing sugars or methylglyoxal (MGO). These results suggest that SME could inhibit protein glycation by a mechanism antioxidant, which could also interfere with the MGO formation. The MGO has been shown that causing the generation of carboxymethyl-lysine (CML) on proteins in vitro [43]. Increased levels of CML have been shown, which is a mediator in diabetic complications [44,45].

Therefore, the present work evaluated if SME treatment prevents the CML accumulation in the retina of streptozotocin-induced diabetic rats (STZ), associated with oxidative and pro-inflammatory responses triggered by the possible AGEs receptor (RAGE) activation. The results showed that treatment with SME decreased CML staining in the ganglion cell layer (GCL), inner nuclear layer (INL), and outer nuclear layer (ONL). Although SME presented higher levels of CML in INL and ONL than aminoguanidine (AG) and without a difference in GCL, the results suggest that SME treatment could inhibit the production of this AGE in the diabetic retina. Furthermore, it was observed that the increase in CML in the diabetic retinal layers was parallel to the increase in RAGE staining levels.

The RAGE up-regulation and AGE accumulation have been associated with retinal damage. One of the consequences of CML–RAGE interaction is the generation of reactive oxygen species (ROS), which, in turn, induces the overexpression of pro-inflammatory genes [46]. In our results, diabetic retinas treated with SME showed a reduction in the CML and RAGE merged (CML/RAGE), mainly in the GCL and INL layers, suggesting that the decreased CML/RAGE could attenuate the downstream activation of the pathways of oxidative stress and inflammatory responses that have been described for the RAGE activation [46].

The oxidative stress induced by AGEs-RAGE is mediated by activating the NADPH oxidases enzymes, which are producers of superoxide anion and are increased in diabetic retinal cells [46,47]. The NADPH oxidases, Nox1/Nox4, contribute to increased ROS, inflammation, and retinal neovascularization [48]. Additionally, the generated superoxide reacts with nitric oxide (NO), producing peroxynitrite, another free radical which participates in the damage of the diabetic retina [49,50]. Elevated NO levels in diabetic retinas are mediated by the augmented activity of the inducible nitric oxide synthase (iNOS), causing an increase in acellular capillaries density, retinal thickening, gliosis, and elevated nitrate/nitrite levels [51]. Here, the results show that SME treatment decreased Nox4 immunostaining in GCL, INL, and ONL compared to STZ. Additionally, compared to AG, SME presented a more significant reduction in Nox4 staining in the three retinal layers.

Regarding the iNOS staining level, there was a decrease in the three retinal layers in the SME group than STZ. When the iNOS staining in AG was compared with STZ, the results showed a more significant decrease than SME in the GCL, INL, and ONL layers, demonstrating that AG is a potent inhibitor of iNOS as has been reported in diabetic retinopathy (DR) [29].

Concerning immunostaining for 3-nitrotyrosine, SME decreased this marker in all three layers compared to STZ. On the other hand, lipid peroxidation and AGEs formation are closely linked in DR [13]. CML is also produced by the oxidation of polyunsaturated fatty acids (PUFAs) [52] found in abundance in the retina. The oxidation reactions of PUFAs lead to the formation of reactive carbonyl species such as malondialdehyde (MDA) that subsequently react with residues of cysteine, histidine, and lysine of proteins, generating relatively stable adducts [53]. Our results demonstrate that CML staining in the layers of the diabetic retina decreased with SME treatment, which is consistent with a decreased lipid peroxidation.

The AGE–RAGE interaction in diabetic retinas is a condition that increases the production of inflammatory factors that, in turn, participate in retinal degeneration. This interaction leads to activation of nuclear NF-κB, which induces the overexpression of pro-inflammatory factors, such as iNOS and the vascular endothelial growth factor (VEGF) [54]. VEGF is a potent mitogenic activator and the central orchestrator of angiogenesis in DR. Nox4 also regulates VEGF expression under conditions of oxidative stress and hyperglycemia [55]. Thus, blocking the AGE–RAGE interaction may be important in attenuating VEGF expression in the pathogenesis of DR.

On the other hand, glial fibrillary acidic protein (GFAP) is a protein present in retinal glial cells (ganglion cells), astrocytes, Müller cells, and microglia, and its increase indicates the formation of neural gliosis in the retina [56]. Therefore, the nuclear translocation of NF-κB and the overexpression of VEGF and GFAP are pro-inflammatory factors related to the pathogenesis of DR [13]. These factors are generated, in turn, by the formation of AGEs and AGEs-RAGE interaction [45,57,58]. Here, it was shown that SME treatment decreased CML-RAGE merged in the layers of diabetic retinas in addition to a reduction in nuclear NF-κB, VEGF, and gliosis. These results suggest that SME could attenuate the inflammatory response by inhibition of the CML–RAGE interaction.

The inflammatory response was also evaluated by staining of S100 calcium-binding protein B (S100B). S100B is a protein that increases by oxidative stress and stimulates the inflammatory response and the neurodegeneration in the DR [58,59]. The results show that S100B staining was distributed in the three retinal layers in STZ. It has been reported that in INL, Müller glial cells are found [58], which could be related to gliosis susceptibility. S100B staining in INL was decreased with SME treatment compared to STZ and AG, suggesting that SME in this layer inhibits the inflammatory response.

In normoglycemic rats, S100B levels in ONL were higher than the STZ groups. Possibly, in normoglycemic rats, the S100B levels could be taking part in the regulation of a membrane-bound guanylate cyclase-based signaling pathway in photoreceptors [60]. In diabetic groups, the decreased S100B levels could produce a loss of phototransduction in the photoreceptor cell, which uses cGMP as a second messenger to couple the light absorption to changes in cation channels’ conductivity in the plasma membrane [61]. Thus, SME could decrease S100B protein levels by their antioxidative properties [62]. However, further studies are required to characterize the function and interaction of S100B in the different retinal layers.

In diabetes mellitus (DM), cell death has been observed in several types of retinal cells, such as ganglion cells, glial cells (Müller cells and astrocytes), and microglia [63]. AGEs/RAGE can induce apoptosis in retinal neuronal cells mediated by increased oxidative stress or activation of pro-inflammatory cytokines [64]. In this study, the results prove that in GCL, INL, and ONL, the apoptosis was attenuated by SME.

## 5. Conclusions

This study showed that SME consumption improved the pathological profile induced by diabetes in the retinal layers of streptozotocin-induced diabetic rats (STZ). In the in vitro study, SME showed antioxidant activity by inhibiting intermediates of the Maillard reaction (fructosamine and di-carbonylic compounds), preventing the formation of AGEs and the depletion of reduced thiol groups in BSA. In the diabetic retina, SME treatment attenuated CML formation, the CML–RAGE interaction, and activation of the pro-inflammatory responses (VEGF, GFAP, S100B, and RAGE). Furthermore, SME induced decreased oxidative stress markers (iNOS, Nox4, and NT) and apoptosis. Finally, the results show that SME has a protective effect on the retinal degeneration of STZ.

## Figures and Tables

**Table 1:** The in vitro effects of spinach methanolic extract (SME) on BSA glycation. Aliquots of reaction mixtures were used to determine AGEs formation, fructosamine level, protein carbonyl, and thiol group contents.

AGEs formation (FIU)				
	Mixture	Glucose	Fructose	Methylglyoxal
**BSA**	-	184.7 ± 29.47	317.3 ±30.73	286.2 ± 31,78
	SME-50	167.9 ± 12.49	295.0 ± 13.71	267.2 ± 11.23
	SME-100	158.4 ± 12.11	281.4 ± 17.45	256.2 ± 12.95
	SME-200	149.1 ± 11.83 *	267.5 ± 18.11 *	243.2 ± 15.01 *
	AG	128.4 ± 13.22 *	250.6 ± 14.65 *	229.1 ± 12.78 *
**Fructosamine formation (nmol/L)**				
	**Mixture**	**Glucose**	**Fructose**	**Methylglyoxal**
**BSA**	-	8.81 ± 1.46	4.55 ± 1.15	0.69 ± 0.21
	SME-50	7.37 ± 1.39	3.76 ± 0.93	0.49 ± 0.19
	SME-100	6.94 ± 0.97	3.11 ± 0.62	0.33 ± 0.17
	SME-200	6.16 ± 0.81 *	2.53 ± 0.53 *	0.28 ± 0.07 */α
	AG	5.58 ± 0.71 *	2.11 ± 0.53 *	0.33 ± 0.05 *
**Carbonyl Groups formation (nmol/mg protein)**				
	**Mixture**	**Glucose**	**Fructose**	**Methylglyoxal**
**BSA**	-	2.57 ±1.46	3.77 ± 0.6	2.12 ± 0.54
	SME-50	2.33 ± 1.39	3.11 ± 0.45	2.76 ± 1.15
	SME-100	2.02 ± 0.97	2.92 ± 0.45	1.82 ± 0.79
	SME-200	1.95 ± 0.8 *	2.84 ± 0.45 *	1.88 ± 0.52 *
	AG	1.40 ± 0.71 *	2.12 ± 0.54 *	1.64 ± 0.33 *
**Reduced thiol groups (nmol/mg)**				
	**Mixture**	**Glucose**	**Fructose**	**Methylglyoxal**
**BSA**	-	0.72 ± 0.14	0.62 ± 0.1	0.66 ± 0.09
	SME-50	0.75 ± 0.09	0.68 ± 0.07	0.74 ± 0.01
	SME-100	0.84 ± 0.1	0.75 ± 0.09	0.83 ± 0.12
	SME-200	0.95 ± 0.14 *	0.84 ± 0.11 *	0.98 ± 0.01 */α
	AG	1.08 ± 0.15 *	0.93 ± 0.08 *	0.87 ± 0.12 *

The SME (50, 100, and 200 mg/mL) were mixed with bovine serum albumin (BSA) and reducing sugars (glucose, fructose) or methylglyoxal. AG = aminoguanidine (1 mg/mL); FIU = fluorescence intensity units. Data are media ± SD, * *p* < 0.05 compared to positive control and α *p* < 0.05 compared to AG.

## Supplementary Materials

The following are available online at https://www.mdpi.com/article/10.3390/antiox10050717/s1, Supplement 1: Chemical structures of main compounds isolated from aerial parts of *S. oleracea* which including several phenolic compounds (flavones, flavonoids, coumarins) carotenoids (lutein and/or zeaxanthin), Supplement 2: A. Formation of fructosamine, di-carbonylic compounds, and AGE molecules by Maillard reaction when incubating bovine serum albumin (BSA) with reducing sugars or with MGO by mon-enzymatic reactions. B. The probable transformations of the reduced thiol group of Cys34 from BSA to Carboxy-S-cys, Carboxymethyl-S-cys, and hemithioacetal induced by MGO and glyoxal are shown as has been proposed in other reports, Suplemment 3: Representative micrographs of immunofluorescent staining with the antibodies for NADPH oxidase 4 (Nox-4).

## Appendix A

**Table A1:** Effect of spinach methanolic extract (SME) in the retina of streptozotocin-induced diabetic rats (STZ).

Integral Optical Density (IOD)			
**CML**			
**Group**	**GCL**	**INL**	**ONL**
NG	2766 ± 657	3002 ± 961	4812 ± 949
STZ	6066 ± 651 *	7978 ± 871 *	7442 ± 732 *
STZ-AG	3772 ± 719 */α	4482 ± 1153 */α	5674 ± 1076 */α
STZ-SME	4318 ± 443 */α	5227 ± 982 */α/β	6534 ± 826 */α/β
**RAGE**			
**Group**	**GCL**	**INL**	**ONL**
NG	2137 ± 527	3093 ± 653	3950 ± 604
STZ	4473 ± 597 *	5502 ± 706 *	5549 ± 721 *
STZ-AG	2998 ± 734 */α	4147 ± 522 */α	4447 ± 461 */α
STZ-SME	3376 ± 584 */α	4391 ± 655 */α	4951 ± 449 */α/β
**CML/RAGE**			
**Group**	**GCL**	**INL**	**ONL**
NG	1369 ± 395	1338 ± 353	2408 ± 324
STZ	3439 ± 483 *	5002 ± 604 *	4489 ± 427 *
STZ-AG	1721 ± 512 */α	2262 ± 351 */α	2776 ± 387 */α
STZ-SME	2253 ± 446 */α/β	2430 ± 485 */α	3234 ± 483 */α/β

Immunostaining values by integrated optical density (IOD, lum/in^2^) for CML, RAGE, and its combination (CML/RAGE) in the retinal layers (GCL, INL, and ONL) of normoglycemic rats (NG), STZ, aminoguanidine-treated STZ (STZ-AG), and STZ rats treated with SME (STZ-SME). Ganglion cell layer (GCL), inner nuclear layer (INL), and outer nuclear layer (ONL). Data show mean ± SD. * *p* < 0.01 compared to NG; α *p* < 0.01 compared to STZ; and β *p* < 0.01 compared to STZ-AG.

**Table A2:** Antioxidant effect of SME on the retina of STZ rats.

% of Cell Positive Immunostaining			
**NADPH-Nox4**			
**Group**	**GCL**	**INL**	**ONL**
NG	19.3 ± 3.9	19.0 ± 3.2	27.5 ± 3.4
STZ	50.7 ± 5.8 *	42.6 ± 5.0 *	50.5 ± 3.6 *
STZ-AG	35.1 ± 4.8* /α	37.8 ± 3.8 */α	47.0 ± 3.6 */α
STZ-SME	31.8 ± 3.7 */α/β	34.5 ± 982 */α/β	41.1 ± 3.4 */α/β
**iNOS**			
**Group**	**GCL**	**INL**	**ONL**
NG	19.2 ± 4.2	18.6 ± 3.3	29.7 ± 3.2
STZ	34.8 ± 6.3 *	31.9 ± 3.7 *	49.9 ± 5.3 *
STZ-AG	24.9 ± 5.0 */α	24.1 ± 5.3 */α	34.9 ± 4.3 */α
STZ-SME	29.8 ± 5.1 */α/β	27.3 ± 3.7 */α/β	45.9 ± 4.0 */α/β
**Nitrotyrosine**			
**Group**	**GCL**	**INL**	**ONL**
NG	19.9 ± 3.9	18.5 ± 3.2	14.2 ± 1.8
STZ	29.3 ± 4.1 *	33.9 ± 4.8 *	52.0 ± 3.5 *
STZ-AG	25.5 ± 3.6 */α	31.5 ± 3.6 *	48.9 ± 3.9 */α
STZ-SME	25.1 ± 4.2 */α	25.6 ± 4.6 */α/β	33.8 ± 2.8 */α/β

Percentage of immunostained cells for NADPH oxidase 4 (Nox-4), inducible nitric oxide synthase (iNOS), and nitrotyrosine (NT) are shown in the retinal layers (GCL, INL, and ONL) of NG, STZ, STZ-AG, and STZ-SME. Data show mean ± SD. * *p* < 0.01 compared to NG; α *p* < 0.01 compared to STZ; and β *p* < 0.01 compared to STZ-AG.

**Table A3:** Effect of SME on the retinal inflammation of STZ rats.

Nuclear NF-kB (%)			
**Group**	**GCL**	**INL**	**ONL**
NG	17.3 ± 2.9	17.1 ± 1.9	15.3 ± 2.1
STZ	42.1 ± 4.4 *	42.4 ± 5.1 *	36.1 ± 3.2 *
STZ-AG	35.3 ± 3.8 */α	39.4 ± 6.6 */α	17.8 ± 2.2 */α
STZ-SME	32.3 ± 3.1 */α/β	27.5 ± 6.9 */α/β	15.0 ± 2.7 α/β
**S100B (IOD)**			
**Group**	**GCL**	**INL**	**ONL**
NG	4356 ± 849	3464 ± 1170	4207 ± 1160
STZ	7721 ± 916 *	7916 ± 1103 *	3317 ± 827 *
STZ-AG	7081 ± 1037 */α	5836 ± 1041 */α	2438 ± 729 */α
STZ-SME	5248 ± 903 */α/β	3862 ± 736 */α/β	2233 ± 735 */α

The percentage nuclear of NF-κB staining and values IOD for S100B in the retinal layers (GCL, INL, and ONL) of NG, STZ, STZ-AG, and STZ-SME groups are shown. Data show mean ± SD. * *p* < 0.01 compared to NG; α *p* < 0.01 compared to STZ; and β *p* < 0.01 compared to STZ-AG.
