# Supplementary material for: Spinach Methanolic Extract Attenuates the Retinal Degeneration in Diabetic Rats
Source: Antioxidants (Basel). 2021 May 3;10(5):717. doi: 10.3390/antiox10050717 (PMC8147642; doi:10.3390/antiox10050717)
Supplement: Supplementary file 1 [file antioxidants-10-00717-s001.zip › antioxidants-1174210-supplementary.pdf]

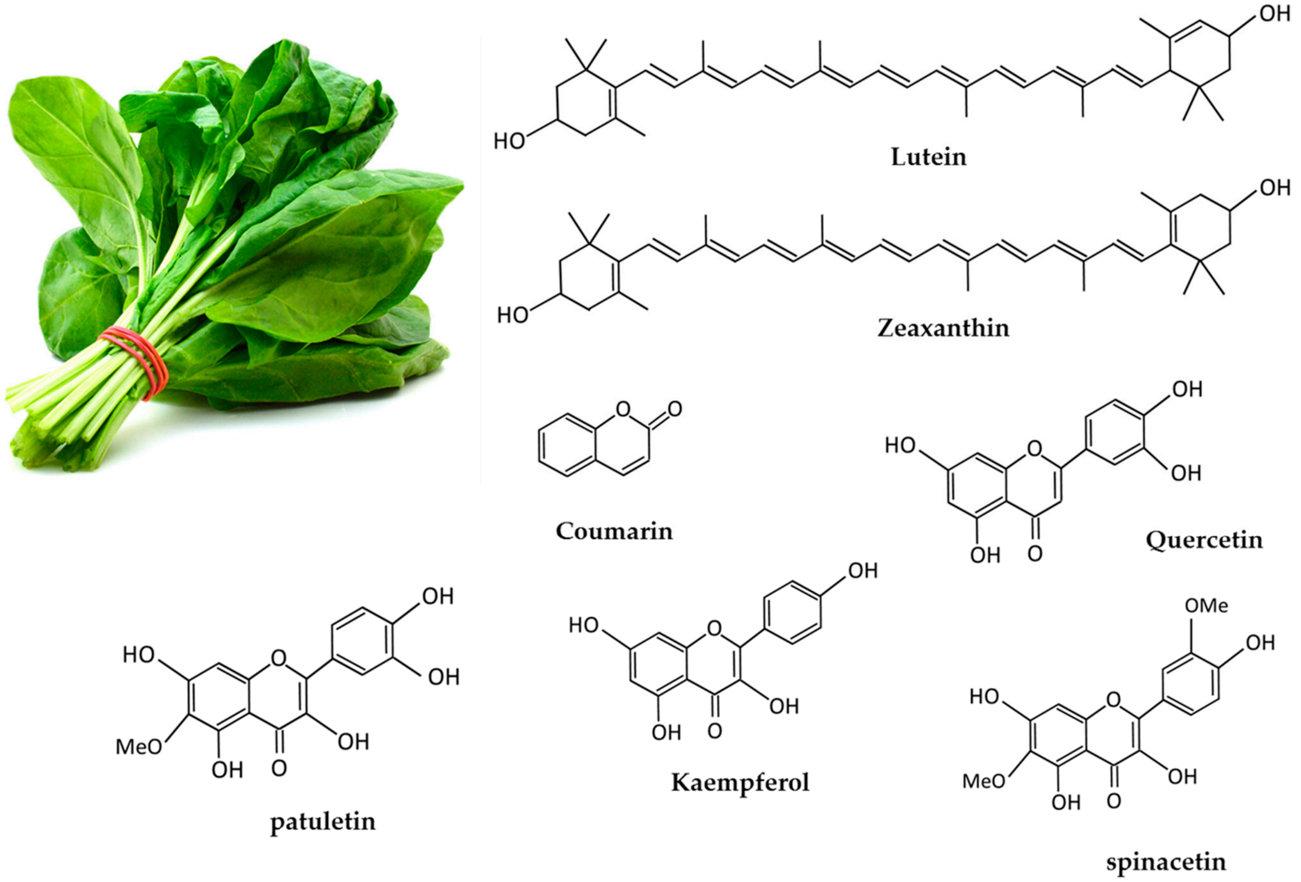

Supplement 1. Chemical structures of main compounds isolated from aerial parts of S. oleracea which including several phenolic compounds (flavones, flavonoids, coumarins) carotenoids (lutein and/or zeaxanthin).

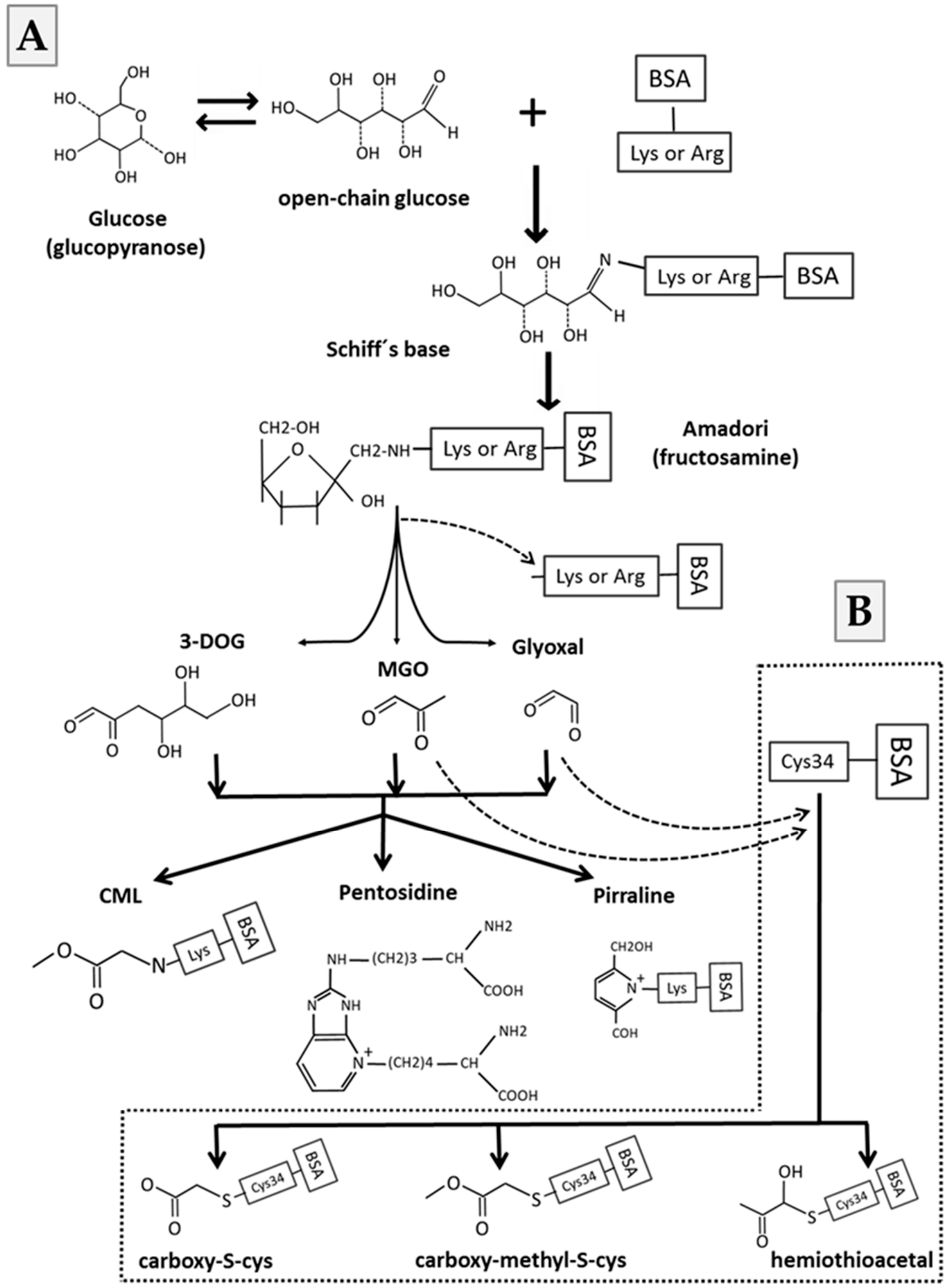

Supplement 2 A. Formation of fructosamine, di-carbonylic compounds, and AGE molecules by Maillard reaction when incubating bovine serum albumin (BSA) with reducing sugars (glucose or fructose) or with MGO by non-enzymatic reactions. The dicarbonyl intermediate compounds as glyoxal, MGO, and 3-DOG are formed by non-oxidative rearrangement and hydrolysis of Amadori products. These carbony compounds lead to AGEs formation as the CML, pentosidine, and pirraline [40,41]. **B.** The probable transformations of the reduced thiol group of Cys34 from BSA to Carboxy-S-cys, Carboxymethyl-S-cys, and hemithioacetal induced by MGO and glyoxal are shown as has been proposed in other reports [40,43].

Abbreviations:

BSA: bovine serum albumin, Lys:lysine, Arg:arginine, 3-DOG: 3-deoxiglucosona, MGO: methyl-glioxal, AGEs: advanced glycation end products, Nε-carboxymethyl lysine: CML, Cys34: cysteine 34, Carboxy-S-cys: carboxy-S-cysteine, Carboxy-S-methyl-cys: carboxymethyl-S-cysteine.

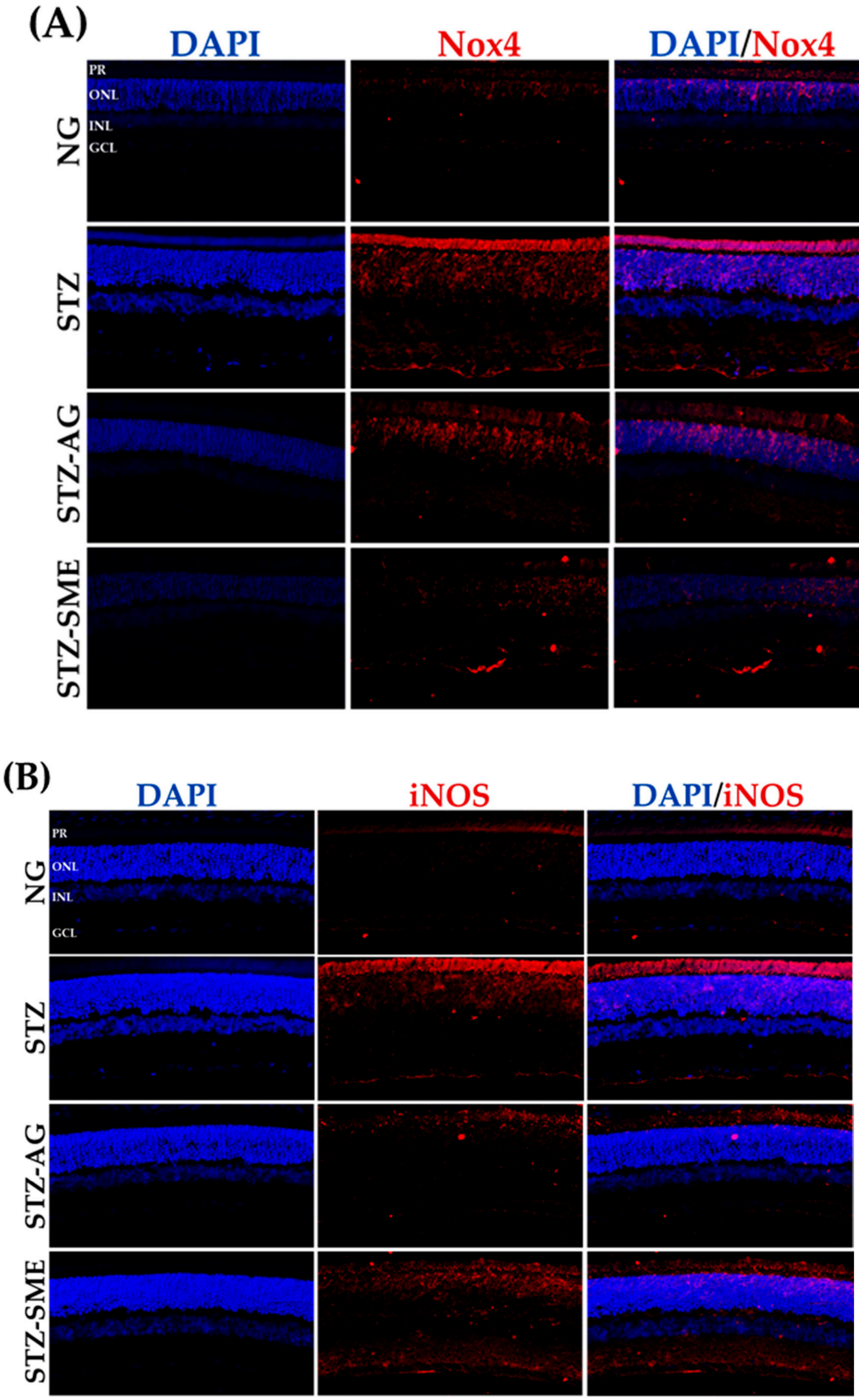
(A)
DAPI
Nox4
DAPI/Nox4
PR
ONL
INL
GCL
NG
STZ
STZ-AG
STZ-SME
(B)
DAPI
iNOS
DAPI/iNOS
PR
ONL
INL
GCL
NG
STZ
STZ-AG
STZ-SME

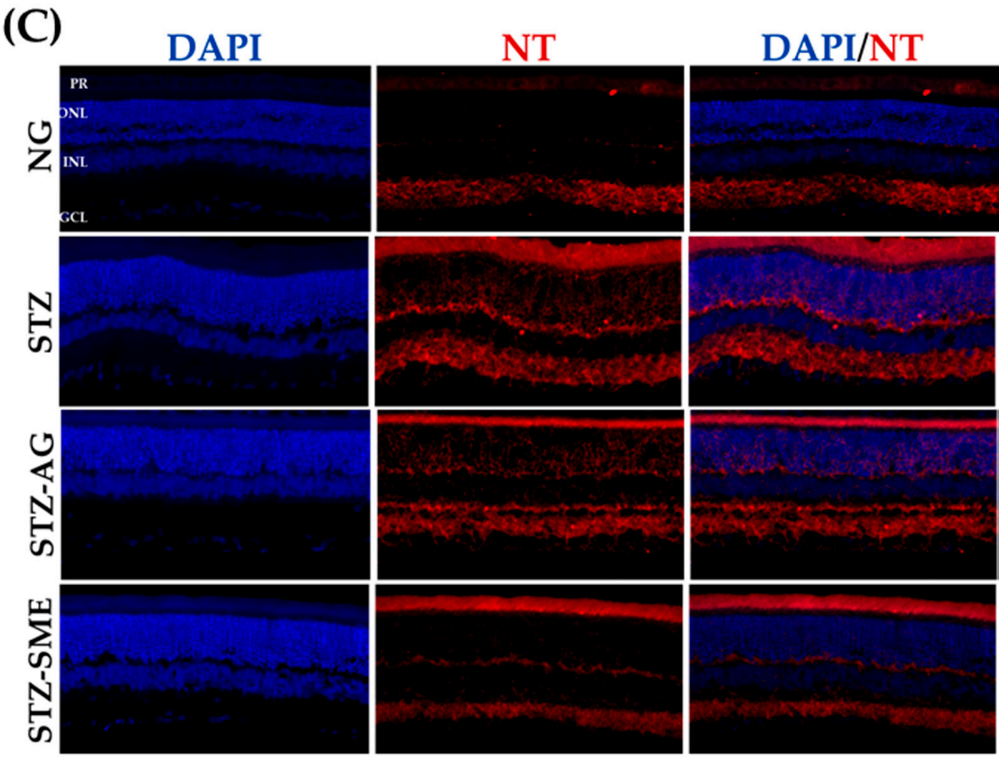

Suplemment 3. Representative micrographs of immunofluorescent staining with the antibodies for NADPH oxidase 4 (Nox-4) (A), inducible nitric oxide synthase (iNOS) (B), and nitrotyrosine (NT) (C) in normoglycemic rats (NG), STZ, treated with aminoguanidine (STZ-AG) and treated with SME (STZ-SME); the nuclei were stained with 4′,6-Diamidine-2′-phenylindole dihydrochloride (DAPI) (blue). Magnification 200×.
